# Considerations of target surface area and the risk of radiosurgical toxicity

**DOI:** 10.1371/journal.pone.0224047

**Published:** 2019-10-21

**Authors:** Strahinja Stojadinovic, Yulong Yan, Andrew Leiker, Chul Ahn, Zabi Wardak, Tu Dan, Lucien Nedzi, Robert Timmerman, Toral Patel, Samuel Barnett, Bruce Mickey, Jeffrey Meyer

**Affiliations:** 1 Department of Radiation Oncology, University of Texas Southwestern Medical Center, Dallas, TX, United States of America; 2 Department of Population and Data Sciences, University of Texas Southwestern Medical Center, Dallas, TX, United States of America; 3 Department of Neurological Surgery, University of Texas Southwestern Medical Center, Dallas, TX, United States of America; 4 Department of Radiation Oncology and Molecular Radiation Sciences, Johns Hopkins University School of Medicine, Baltimore, MD, United States of America; MD Anderson Cancer Center, UNITED STATES

## Abstract

**Objective:**

The goal of this study was to explore conceptual benefits of characterizing delineated target volumes based on surface area and to utilize the concept for assessing risk of therapeutic toxicity in radiosurgery.

**Methods and materials:**

Four computer-generated targets, a sphere, a cylinder, an ellipsoid and a box, were designed for two distinct scenarios. In the first scenario, all targets had identical volumes, and in the second one, all targets had identical surface areas. High quality stereotactic radiosurgery plans with at least 95% target coverage and selectivity were created for each target in both scenarios. Normal brain volumes V_12Gy_, V_14Gy_ and V_16Gy_ corresponding to received dose of 12 Gy, 14 Gy and 16 Gy, respectively, were computed and analyzed. Additionally, V_12Gy_ and V_14Gy_ volumes and values for seven prospective toxicity variables were recorded for 100 meningioma patients after Gamma Knife radiosurgery. Multivariable stepwise linear regression and best subset linear regression analyses were performed in two statistical software packages, *SAS/STAT* and *R*, respectively.

**Results:**

In a phantom study, for the constant volume targets, the volumes of 12 Gy, 14 Gy and 16 Gy isodose clouds were the lowest for the spherical target as an expected corollary of the isoperimetric inequality. For the constant surface area targets, a conventional wisdom is confirmed, as the target volume increases the corresponding volumes V_12Gy_, V_14Gy_ and V_16Gy_ also increase. In the 100-meningioma patient cohort, the best univariate model featured tumor surface area as the most significantly associated variable with both V_12Gy_ and V_14Gy_ volumes, corresponding to the adjusted *R*^*2*^ values of 0.82 and 0.77, respectively. Two statistical methods converged to matching multivariable models.

**Conclusions:**

In a univariate model, target surface area is a better predictor of spilled dose to normal tissue than target largest dimension or target volume itself. In complex multivariate models, target surface area is an independent variable for modeling radiosurgical normal tissue toxicity risk.

## Introduction

Surface area is an essential building block for numerous theoretical and technological concepts in mathematics, physics, chemistry, biology, cosmology, and other natural sciences. The motive for this work was to employ the concept in radiation therapy setting and to evaluate risk of radiosurgical toxicity from an unexplored vantage point of target surface area.

In radiation oncology, the ICRU standards for therapeutic use of radiation[1–7] necessitate stringent definition of target volumes delineated on geometrically accurate volumetric imaging datasets. The radiotherapy is administered by strictly following radiation dose prescription. Prescription selection is, in part, governed by efforts to avoid predictable radiotherapy toxicities, by restricting undesired dose to the organs at risk (OARs) in accord with commonly accepted normal tissue tolerance limits [8–14]. A goal is to cover all or a substantial proportion of the target volume with the prescription dose. In treatment planning process, the volumes of three-dimensional objects are defined via slice by slice contouring in form of closed planar curves. An ideal treatment plan has a prescription isodose line perfectly matching target contours in all slices. Prescription dose can thus be envisioned as an isodose cloud that conforms to the surface area of the target. In practice, a departure from an ideal dose distribution is dictated by treatment delivery limitations and patient specific geometry which leads to toxicity tradeoffs. The RTOG 90–05 protocol[15] for stereotactic radiosurgery is a prime example of prescription dose limited by toxicity data derived from representing target volumes by the largest linear dimension. An alternate way of characterizing target volume is the volume itself, with the corresponding prescription specified as a percent volume coverage[5]. There is yet another fundamental alternative, volumes of three-dimensional objects can be characterized in terms of two-dimensional surface area. This is linked to a mathematics problem spanning to the origins of geometry, i.e., the classical isoperimetric problem[16]. The goal is to determine a closed plane curve of a given perimeter which encloses the greatest area. The solution to this problem is a circle. In contemporary mathematical terms, in Euclidian spaces, the isoperimetric inequality states that a sphere has the smallest surface area per given volume.

Thus far, the mathematical concept of surface area has not been utilized in modern radiotherapy treatment planning as target surface area is neither measured nor considered for plan quality evaluations and toxicity assessments. This manuscript is an effort in exploring conceptual benefits of representing delineated target volumes based on surface area. The concept was validated in a stereotactic radiosurgery setting utilizing Leksell GammaPlan (LGP) as the treatment planning platform.

## Methods and materials

The volume of normal tissue around a tumor, analogous to onion layers, can be imagined as rinds of tissue where each rind corresponds to a specific isodose line increment. The volume of the rind of tissue *V*_*rind*_ of thickness *d* surrounding spherical target of radius *r* is equal to the volume of a larger sphere *V*(*r+d*) minus the internal sphere volume *V*(*r*):

$$\begin{array}{l} V_{rind}=V(r+d)-V(r)=\frac{4\pi }{3}\left[{\left(r+d\right)}^{3}-r^{3}\right]=4\pi r^{2}\cdot d+4\pi r\cdot d^{2}+\frac{4\pi }{3}\cdot d^{3}\\ \;=4\pi r^{2}\cdot \left(d+\frac{d^{2}}{r}+\frac{d^{3}}{3r^{2}}\right)\overset{d\ll r}{\approx }4\pi r^{2}\cdot d \end{array}$$ (1)

If *d* is much smaller than *r*, i.e., *d*/*r* is small, the above equation in linear approximation is equal to *4πr*^*2*^∙*d*, which is the surface area of the original sphere multiplied by the thickness *d*. Intuitively, the layers around tumor are associated with possible radiotherapy treatment complications. Thus, the working hypothesis is that target surface area is a toxicity risk predictor in radiosurgery.

### Phantom study

A radiotherapy treatment planning feature crucial for this study is the ability to measure surface area of delineated targets and critical structures. This requirement is quite basic yet the feature is nonexistent in contemporary commercial treatment planning systems. For that reason, an in-house multifunction software *DICOMan*^*TX*^ [17, 18] was utilized to create a high-resolution spherical phantom and target objects in DICOM format. The numerical methods used for computing surface area and volume of contoured objects are beyond the scope of this report, however, the algorithm was validated on geometric objects with well-known analytical solutions. The virtual spherical phantom was made identical to a 16 cm diameter Leksell Dosimetry Phantom used for absorbed dose and dose rate measurements for Gamma Knife units. Two sets of targets were created next. Each target set included a sphere, a cylinder, an ellipsoid and a box. The first group, a constant volume set, included targets of identical volumes. The second group, a constant surface area set, included targets with identical surface areas. The dimensions of the phantom and the objects are summarized in Table 1.

**Table 1:** Geometric dimensions of phantom and objects used for treatment planning.

LGP Plans	Object	Dimensions [mm]	Volume [cm^3^]	Surface Area [cm^2^]
Phantom	Leksell Dosimetry Phantom	Diameter: *d* = 160.0	2144	804.3
Constant Volume	Sphere	Radius: *r* = 14.96	14.0	28.1
	Cylinder	Radius *r* = 14.0, Height *h* = 22.8	14.0	32.4
	Ellipsoid	Axis: *a* = 17.42, *b* = 8.0, *c* = 24.0	14.0	33.5
	Box	Side *a* = 24.11	14.0	34.9
Constant Surface Area	Sphere	Radius *r* = 14.96	14.0	28.1
	Cylinder	Radius *r* = 12.63, Height *h* = 22.8	11.4	28.1
	Ellipsoid	Axis: *a* = 15.0, *b* = 8.0, *c* = 22.48	11.3	28.1
	Box	Side *a* = 21.65	10.1	28.1

The phantom and the objects were exported from *DICOMan*^*TX*^ to Leksell GammaPlan (LGP) version 11.03. The phantom was delineated utilizing the LGP image-based segmentation tool which provided automatic skull definition. Every object was centrally placed inside of the spherical phantom and designated a target structure. The largest cardinal axis for every target was oriented along the z-axis, i.e., along Gamma Knife in-and-out direction. Such target placement takes advantage of the machine design and source arrangement which results in the sharpest beam penumbra in z-plane.

The aim was to develop eight individual radiosurgery plans of equivalent plan quality. The treatment planning goals were set at a minimum 95% target coverage accompanied with at least 95% selectivity as defined by Paddick[19]. However, plans of equal conformity may inherently, due to diverse isocenter placements, exhibit strikingly different dose falloff characteristics. The gradient index[20], defined as the volume of half the prescription isodose to the volume of the prescription isodose, is designed to further differentiate between plans of comparable conformity. Therefore, the gradient index of less than 3 was imposed as an additional constraint for achieving equivalent plans. The prescribed dose for every target was 18 Gy to 50% isodose line. For every target, all the shots were manually placed inside target contours. The inverse planning optimization with operators’ fine-tuning assistance was used iteratively to meet or exceed the coverage, selectivity and gradient index constraints.

Ideally, clinically recorded long term toxicity data would provide benchmark metrics in any analysis identifying significant outcome variables. In reality, published studies differ in many aspects including prescription dose, definition of volume, completeness of follow up, definition and evidence of radiation toxicity. This information is not available for a phantom study. However, treatment plan toxicity risks can be evaluated as a volume of surrounding normal tissue receiving undesired spilled dose from the neighboring treatment site(s). A normal brain volume, uninvolved by tumor, receiving a dose of 12 Gy or higher is commonly considered as a benchmark complication predictor for a large number of conditions[21]. In addition, the volume of brain receiving ≥12 Gy has been shown to correlate with both the incidence of radiation necrosis and asymptomatic radiologic changes[12]. For that reason, the normal brain volumes V_12Gy_, V_14Gy_ and V_16Gy_ corresponding to received dose of 12 Gy, 14 Gy and 16 Gy, respectively, represented a measure of treatment toxicity risks in this study. In particular, the V_12Gy_, V_14Gy_ and V_16Gy_ brain volumes for a spherical target were used as reference or normalization values for toxicity risk comparisons between all phantom plans.

### Clinical study

Ultimately, an a priori sample size of 100 anonymized clinical patients were selected for tumor surface area measurements utilizing *DICOMan*^*TX*^ software and for statistical evaluations with *SAS/STAT* and *R* software. The selection criteria were simple and included only single fraction meningioma patients treated with a dose of 15 Gy prescribed to 50% isodose line at the periphery of the target. The 15 Gy was selected as this prescription dose was used most frequently within institutional database. These treatments involved de novo patients as well as patients who had residual disease or recurrence after surgical resection. The rationale for including just one prescription dose was to achieve evaluation of equivalent clinical conditions. Otherwise, the prescribed dose differences would need to be accounted by scaling or renormalizing the plans, which would be different from the actual delivered plans. One hundred patients met the inclusion criteria out of 175 Gamma Knife patients treated from November 2011 to July 2018. The 100-patient cohort comprised of 77% female and 23% male patients with a median age of 60.1 years (range 21 to 92 years). Based on the World Health Organization (WHO) classification scheme for meningiomas, 87% of patients were Grade 1, 11% Grade 2 and 2% Grade 3. Radiological features of contrast enhanced MRI images were used for diagnosis determination in 63 patients, while the remaining 37 patients had both histopathological and imaging evaluations. The retrospective statistical studies did not include any interaction or intervention with human subjects and the anonymized data did not include any identifiable patient information. As the data had already been obtained through a standard clinical practice, the research ethics committee or institutional review board approval was not required.

Two different software packages and seven variables were used for statistical investigations. The studied variables included target surface area, target volume, target largest linear dimension, coverage, selectivity, gradient index as well as number of shots or isocenters. Of particular interest was inclusion of target surface area as novel and uncharted variable. Univariate and multivariable linear regression evaluations were performed with *SAS/STAT version 9*.*4* (SAS Institute, Cary, NC)[22]. Forward and backward stepwise linear regression analyses were conducted to identify significant variables associated with V_12Gy_ and V_14Gy_ volumes. Next, a statistical computing platform *R version 3*.*5*.*1* aka *Feather Spray* (RStudio version 1.1.456, RStudio, Inc., Boston, MA)[23] was used to determine best variable subsets relating to V_12Gy_ and V_14Gy_ volumes by utilizing the adjusted coefficient of determination *R*^*2*^. An adjusted coefficient of determination, in contrast to an unadjusted value, penalizes the model for further variable insertion. Comparable models were ranked based on increasing value of adjusted *R*^*2*^ with a maximum value of 1 indicating perfect correlation between linear fit and data. The simplest model is one variable model, following is adding one more variable to the assortment and so forth. The analysis included all variables, however, for simplicity and clarity, the presented results were limited to the best two models for a given number of variables.

## Results

### Phantom study results

*DICOMan*^*TX*^ software was used to create *DICOM* objects in this study. The phantom and two sets of targets, one set of identical volumes and another set of identical surface areas were imported to Leksell GammaPlan. On average, LGP measured target volumes in Table 2 were within 1.1% agreement relative to *DICOMan*^*TX*^ values, with the greatest outlier of 2.6% for the smallest target volume. These differences are expected since there is no universally adopted or recommended method of determining volumes from contours, consequently the proprietary algorithm differences yield slightly different values.

**Table 2:** Attained treatment planning plan quality valuations of coverage, selectivity and gradient index.

LGP Plans	Object	Coverage [%]	Selectivity [%]	Gradient Index	V_TARGET_ [cm^3^]	%ΔV_OBJECT_ [%]
Constant Volume	Sphere	99	98	2.62	14.0	99.1
	Cylinder	95	96	2.76	14.0	99.0
	Ellipsoid	97	97	2.99	14.0	99.0
	Box	95	96	2.91	13.9	98.5
Constant Surface Area	Sphere	99	98	2.62	14.0	99.9
	Cylinder	95	96	2.86	11.3	98.1
	Ellipsoid	96	97	2.99	11.3	99.9
	Box	95	95	2.92	9.8	97.4

V_TARGET_ designates an object volume in cubic centimeters determined by Leksell GammaPlan (LGP). %ΔV_OBJECT_ is the percent volume agreement between volumes created by *DICOMan*^*TX*^ relative to LGP measured values.

Consistent high-grade plan quality was imposed to support meaningful comparisons between the plans. As listed in Table 2, all treatment plans fulfilled or surpassed the planning requirement goals of 95% coverage and 95% selectivity or better. Furthermore, a gradient index value of 3 or less for every treatment plan was also achieved. The best coverage, selectivity and gradient index was obtained for a spherical target taking full advantage of the Gamma Knife machine design with 192 ^60^Co sources distributed in a semispherical halo.

**Fig 1:**
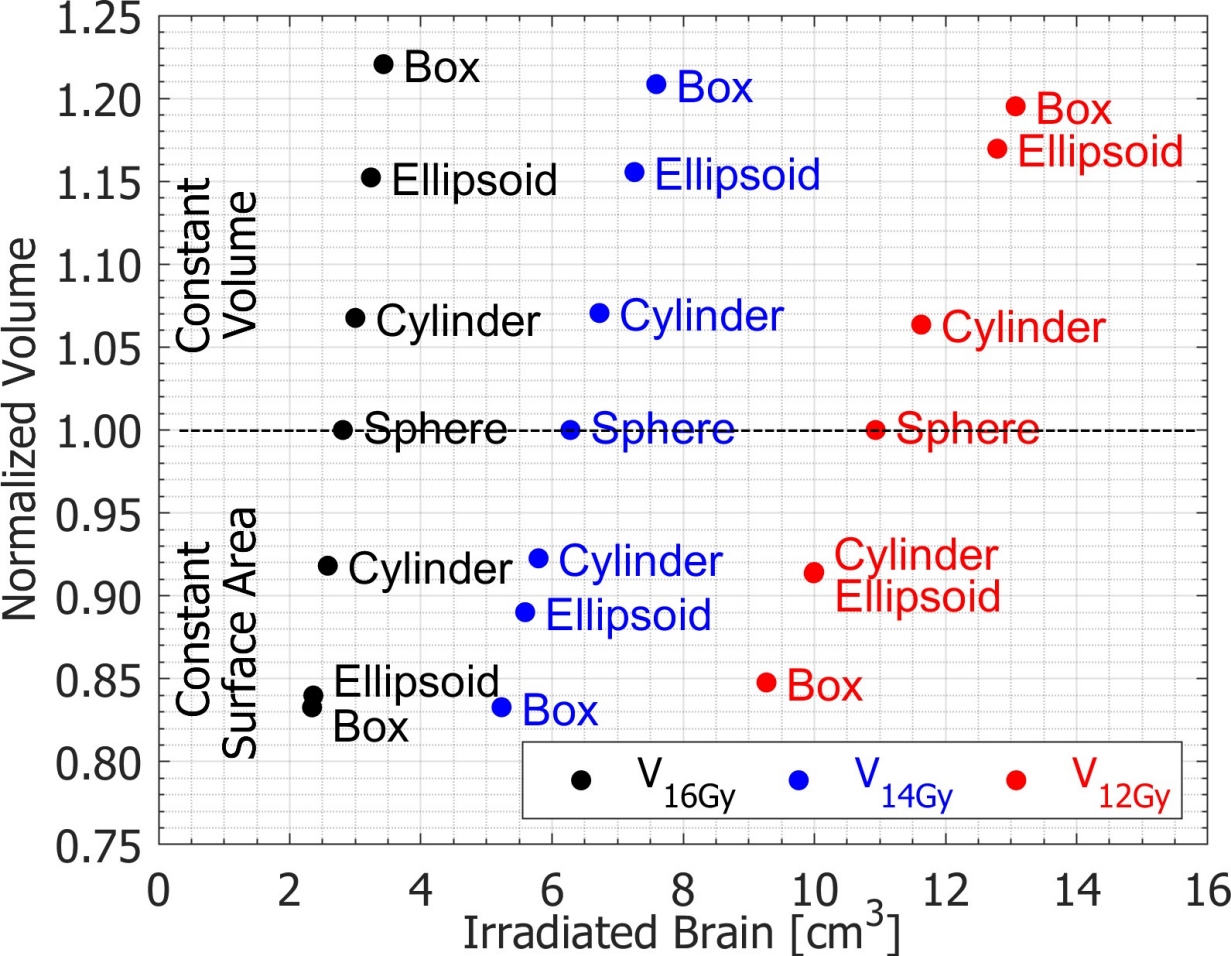
Normal brain volumes of unwanted spilled dose for phantom plans normalized to a reference spherical target plan.

Treatment plans were compared in terms of the volume of undesired spilled dose to the normal brain. For the constant volume targets in Table 3, the volumes of 12 Gy, 14 Gy and 16 Gy isodose clouds were the lowest for the spherical target. In addition, for all constant volume targets, the volumes of unwanted spilled dose around a target normalized to the spherical target baseline values (V_12Gy_)_Object_/(V_12Gy_)_Sphere_, (V_14Gy_)_Object_/(V_14Gy_)_Sphere_ and (V_16Gy_)_Object_/(V_16Gy_)_Sphere_ showed increased ratios for all isodose levels observed, as indicated in Table 3 and Fig 1. For instance, the 16 Gy isodose cloud for the box target was 22% larger compared to the irradiated normal brain volume for spherical target of the same volume.

**Table 3:** Phantom study volumes.

LGP Plans	Object	Brain V_12Gy_ [cm^3^]	(V_12Gy_)_Object_/ (V_12Gy_)_Sphere_	Brain V_14Gy_ [cm^3^]	(V_14Gy_)_Object_/ (V_14Gy_)_Sphere_	Brain V_16Gy_ [cm^3^]	(V_16Gy_)_Object_/ (V_16Gy_)_Sphere_
Constant Volume	Sphere	10.94	1.00	6.28	1.00	2.81	1.00
	Cylinder	11.63	1.06	6.72	1.07	3.00	1.07
	Ellipsoid	12.79	1.17	7.26	1.16	3.24	1.15
	Box	13.07	1.20	7.59	1.21	3.43	1.22
Constant Surface Area	Sphere	10.94	1.00	6.28	1.00	2.81	1.00
	Cylinder	9.99	0.91	5.79	0.92	2.58	0.92
	Ellipsoid	10.00	0.91	5.59	0.89	2.36	0.84
	Box	9.27	0.85	5.23	0.83	2.34	0.83

V_12Gy_, V_14Gy_, V_16Gy_ denote normal brain volumes receiving dose of 12 Gy, 14 Gy and 16 Gy, respectively, after subtraction of the corresponding target volumes. (V_12Gy_)_Object_/(V_12Gy_)_Sphere_, (V_14Gy_)_Object_/(V_14Gy_)_Sphere_ and (V_16Gy_)_Object_/(V_16Gy_)_Sphere_ represent volume ratios of unwanted spilled dose around a target normalized to the reference spherical target baseline values.

For the constant surface area targets, the volumes of 12 Gy, 14 Gy and 16 Gy isodose clouds were the highest for the spherical target, see Table 3. Furthermore, for all constant surface area targets, the corresponding volumes of spilled dose around a target normalized to the spherical target baseline values (V_12Gy_)_Object_/(V_12Gy_)_Sphere_, (V_14Gy_)_Object_/(V_14Gy_)_Sphere_ and (V_16Gy_)_Object_/(V_16Gy_)_Sphere_ displayed decreased ratios for all isodose levels observed, as shown in Table 3 and Fig 1. For example, the ellipsoid target exhibited 16% smaller 16 Gy volume of spilled dose to normal brain relative to the spherical target of the same surface area.

Fig 1 is simply a visual illustration of data given in Table 3. In the upper part of the graph, when all targets have identical volumes, as target surface area increases, the volume of spilled dose to normal brain also increases. Note that the spherical target has the smallest surface area as indicated in Table 1. In the lower part of the graph, when all targets have identical surface areas, as target volume decreases, the volume of spilled dose to normal brain also decreases. In this case, the spherical target has the largest volume as shown in Table 1.

### Clinical study results

Univariate and multivariate linear regression was performed on 100 meningioma patients as described in the methods section. Each patient received a prescribed therapeutic dose of 15 Gy to 50% isodose line. The median tumor volume was 4.0 cm^3^ (range 0.4 to 20.3 cm^3^) and the corresponding median tumor surface area was 19.0 cm^2^ (range 2.8 to 96.6 cm^2^).

The simplest models, derived by univariate linear regression analysis demonstrated that tumor surface area, tumor largest dimension and tumor volume are significantly associated with both V_12Gy_ and V_14Gy_ volumes. The univariate model results are presented in Table 4. The best univariate model featured tumor surface area as the most significantly associated variable with both V_12Gy_ and V_14Gy_ volumes. This is in accord with the highest corresponding adjusted *R*^*2*^ values of 0.82 for V_12Gy_ and 0.77 for V_14Gy_. The second-best single variable model was tumor largest dimension, with the adjusted *R*^*2*^ values of 0.75 and 0.73, corresponding to V_12Gy_ and V_14Gy_ volumes, respectively. Note that the tumor largest dimension denotes measurements in any of three cardinal views, i.e., axial, coronal or sagittal plane, not the true diagonal largest dimension in a volume.

**Table 4:** Univariate linear regression analysis for 100 Gamma Knife meningioma patients.

Outcome	N	Predictor	Estimate ± Standard Error	P-Value	95% Confidence Intervals	Adj*R*^*2*^
V_12Gy_	100	Intercept	-0.2173±0.2546	0.3954	(-0.7225, 0.2879)	0.819
	100	Surface Area	0.2147±0.0101	2.3857·10^−38^	(0.1946, 0.2349)	
	100	Intercept	-3.5751±0.4765	2.8680·10^−11^	(-4.5207, -2.6295)	0.747
	100	Largest Dimension	2.6840±0.1566	2.9679·10^−31^	(2.3733, 2.9947)	
	100	Intercept	0.2509±0.3194	0.4340	(-0.3829, 0.8846)	0.700
	100	Volume	0.8975±0.0589	1.3324·10^−27^	(0.7806, 1.0144)	
V_14Gy_	100	Intercept	-0.2263±0.1753	0.1998	(-0.5741, 0.1216)	0.768
	100	Surface Area	0.1267±0.0070	4.2918·10^−33^	(0.1128, 0.1405)	
	100	Intercept	-2.3036±0.2983	9.9079·10^−12^	(-2.8956, -1.7116)	0.733
	100	Largest Dimension	1.6181±0.0980	4.7014·10^−30^	(1.4235, 1.8126)	
	100	Intercept	0.1809±0.2308	0.4351	(-0.2771, 0.6389)	0.578
	100	Volume	0.4969±0.0426	2.9359·10^−20^	(0.4125, 0.5814)	

The results of *SAS/STAT* forward and backward stepwise linear regression analysis are summarized in Table 5. Tumor surface area (*p*<0.0001), volume (p<0.0001), selectivity (p<0.0001), gradient index (p = 0.0055) and number of shots (p = 0.0003) were significantly associated with V_12Gy_ volume. Tumor surface area, volume, and gradient index and number of shots were positively associated with V_12Gy_ volume while selectivity was negatively associated with V_12Gy_ volume. Tumor largest dimension and coverage were not significantly associated with V_12Gy_ volume. In the same way, tumor surface area (p = 0.0002), volume (p<0.0001), selectivity (p<0.0001), and tumor largest dimension (p<0.0001) were significantly associated with V_14Gy_ volume. Tumor surface area, volume, and largest dimension were positively associated with V_14Gy_ volume while selectivity was negatively associated with V_14Gy_ volume. Gradient index, number of shots and coverage were not significantly associated with V_14Gy_ volume.

**Table 5:** *SAS/STAT* stepwise linear regression analysis for 100 Gamma Knife meningioma patients.

Outcome	N	Predictor	Estimate ± Standard Error	P-Value	95% Confidence Intervals
V_12Gy_	100	Intercept	2.3754±1.5326	0.1245	(-0.6676, 5.4184)
	100	Surface Area	0.0678±0.0143	<0.0001	(0.0394, 0.0962)
	100	Volume	0.6333±0.0603	<0.0001	(0.5135, 0.7531)
	100	Selectivity	-0.0958±0.0106	<0.0001	(-0.1169, -0.0747)
	100	Gradient Index	1.3433±0.4724	0.0055	(0.4053, 2.2813)
	100	Number of Shots	0.0448±0.0120	0.0003	(0.0209, 0.0687)
V_14Gy_	100	Intercept	3.7505±0.6705	<0.0001	(2.4195, 5.0815)
	100	Surface Area	0.0417±0.0108	0.0002	(0.0203, 0.0632)
	100	Volume	0.2560±0.0446	<0.0001	(0.1675, 0.3445)
	100	Selectivity	-0.0655±0.0081	<0.0001	(-0.0815, -0.0495)
	100	Largest Dimension	0.4963±0.1183	<0.0001	(0.2614, 0.7312)

Tumor surface area, volume, selectivity, gradient index and number of shots were significantly associated with V_12Gy_ volume. Tumor surface area, volume, selectivity and largest dimension were significantly associated with V_14Gy_ volume.

**Fig 2:**
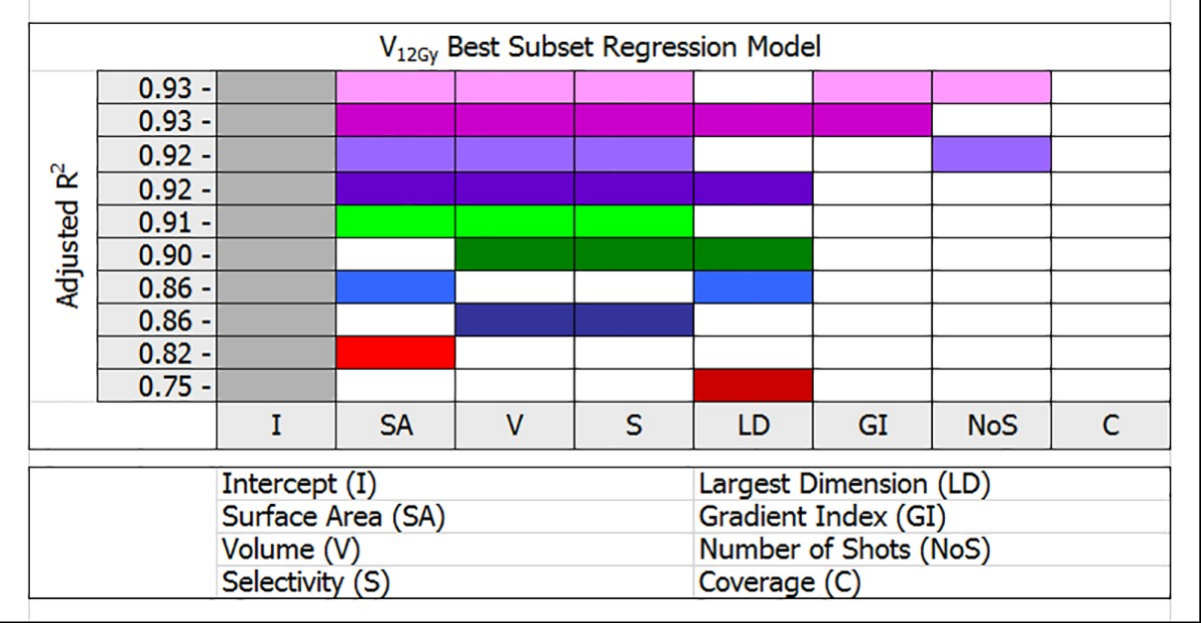
*R* multivariable V_12Gy_ best subset linear regression analysis for 100 Gamma Knife meningioma patients. The color-coded parts in each row indicate number of variables used to model V_12Gy_ volume. Best two models for a given number of variables are shown.

**Fig 3:**
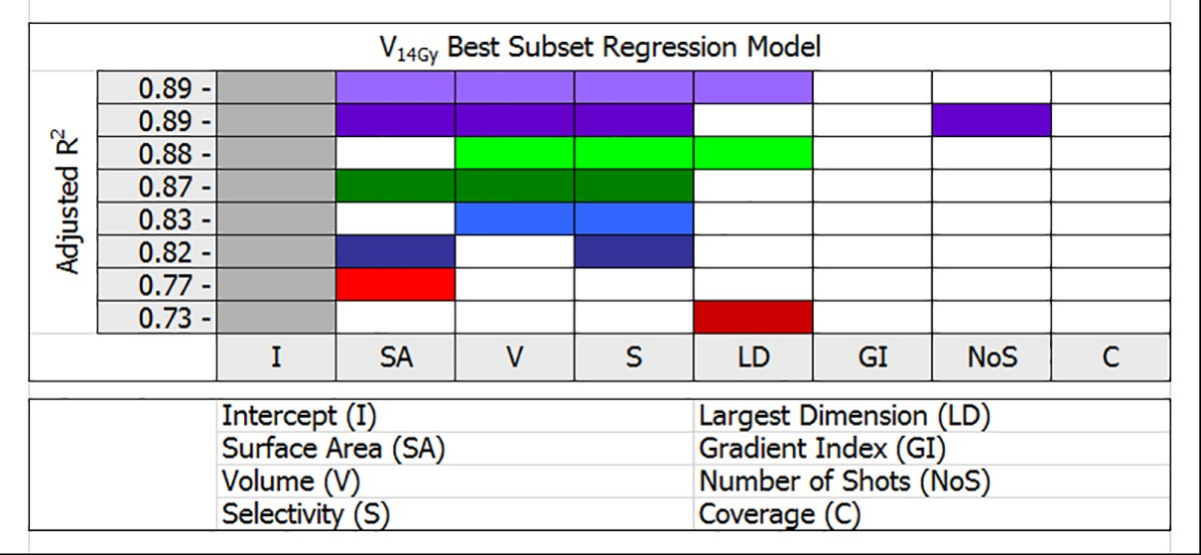
*R* multivariable V_14Gy_ best subset linear regression analysis for 100 Gamma Knife meningioma patients. The color-coded parts in each row indicate number of variables used to model V_14Gy_ volume. Best two models for a given number of variables are shown.

The findings of *R* best subset linear regression analysis limited to the best two models for a given number variables are presented in Figs 2 and 3. Further evaluation of models with two or more variables resulted in improved adjusted *R*^*2*^ values indicating better but more complex models relative to a single variable model. For instance, in Fig 2, there are two models of V_12Gy_ volume with five variables and an equal adjusted *R*^*2*^ value of 0.93. These two models can account for 93% variance in V_12Gy_ volume with four shared variables: tumor surface area, volume, selectivity and gradient index, while the fifth variable is either number of shots or tumor largest dimension. This is in excellent agreement with the stepwise linear regression result for V_12Gy_ in Table 5, with a tie-break observation that the second model included tumor largest dimension as a variable which did not emerge as statistically significant. In the same way, in Fig 3, an equal adjusted *R*^*2*^ value of 0.89 was associated with two models of V_14Gy_ volume involving four variables. In this case three mutual variables are tumor surface area, volume and selectivity, while the fourth variable is either tumor largest dimension or number of shots. Compared to the V_14Gy_ result in Table 5, two statistical methods again converged to the same answers, once more a tie-break decision based on observation that number of shots was not a statistically significant variable in this scenario.

**Fig 4:**
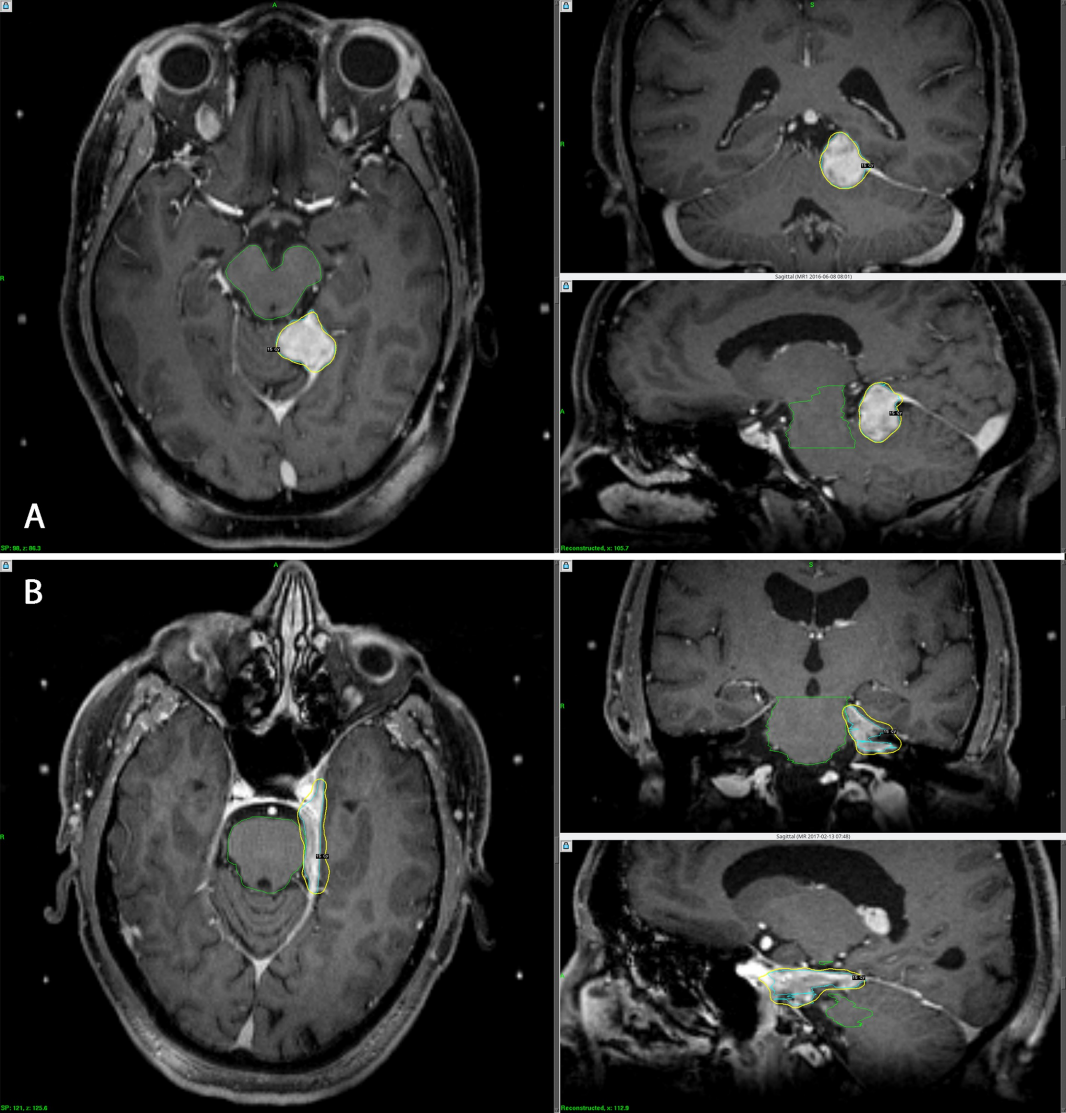
An example of two radiosurgery plans with equivalent traditional treatment metrics yet notably different normal tissue complication probabilities. **Fig 4A. Left tentorial meningioma (above). Fig 4B. Left cavernous sinus meningioma (below).** Both tumors had identical volumes of 4.6 cm^3^ treated with identical dose of 15 Gy prescribed to 50% isodose line. However, the surface area of cavernous sinus meningioma was approximately two times larger relative to the tentorial meningioma, 34.9 cm^2^ versus 16.9 cm^2^, respectively. In turn, the undesired spilled dose to surrounding normal tissue was very different. Accordingly, the risk of an adverse effect is likely to be very different for these two patients.

Fig 4 demonstrates a clinical example of two lesions with the same volumes but different surface areas. One target lesion was in the tentorium while the other abutted the cavernous sinus. Both tumors had identical volumes of 4.6 cm^3^ treated with identical prescription dose of 15 Gy prescribed to 50% isodose line at the periphery of the target. However, the normal tissue toxicity risk is likely to be very different for these two patients due to differences in spilled dose volumes. The surface area of cavernous sinus meningioma was approximately two times larger relative to the tentorial meningioma, 34.9 cm^2^ versus 16.9 cm^2^, respectively. Consequently, the undesired dose spillage to surrounding normal tissue was also very different. For example, V_14Gy_ was 5.4 cm^3^ for the cavernous sinus target while it was 1.1 cm^3^ for the tentorial target, i.e., approximately 5 times larger normal tissue volume was irradiated. Toxicity increases rapidly once the volume of the brain exposed to >12 Gy is >5–10 cm^3^[12]. This case reveals that target surface area offers valuable insight why two patients with equal tumor volumes and equivalent traditional treatment parameters could still exhibit quite different treatment reactions. As target surface area is independent of plan quality, combining surface area with other commonly used metrics, additional risk stratification can be assessed prior to treatment planning as part of the initial counseling of the patient.

## Discussion and conclusions

Intrinsic properties of surface area emerged as fundamental features in numerous scientific settings. In chemistry, the rate of a chemical reaction increases as the surface area of a substance increases[24]. In biology, the efficiency of digestive absorption is linked to the surface area of microvilli[25]. In mathematics, the applications of surface integrals[26] portray physics phenomena of electromagnetism[27]. In cosmology, the entropy of a black hole is proportional to its surface area[28].

Another instance can be added now to the aforementioned examples. In radiotherapy, surface area of a target determines how much normal tissue is exposed to radiation. Target surface area is a fundamental way of characterizing the “size” of a tumor and thus the risk of toxicity to the surrounding brain. The most common metrics currently in use, e.g., target largest dimension and volume, have never been prospectively validated as predictors of toxicity, but are routinely used to make clinical decisions regarding prescribed dose. A univariate model demonstrated that target surface area is a better predictor of spilled dose to normal tissue than target largest dimension or target volume itself. Furthermore, in more complex multivariate models, target surface area maintains the status of a statistically significant variable although it may not necessarily be as a leading term. This reveals that target surface area could be a useful supplement to currently used metrics to further stratify risks of toxicity. The utilization of two different statistical software packages for clinical data analysis provided essential insight for modeling decisions and consequent interpretations. *SAS/STAT* was used to identify statistically significant predictors of normal brain dose. *R* was used to study relative contribution of variables for a given model in an effort to identify clinically applicable set of parameters as a compromise of simplicity versus greatest predictive value.

A phantom study was designed and used as proof of concept and to provide unique perspective on possible treatment toxicities from surface area vantage point. Note that the plan for spherical target is in fact the same plan belonging to both constant volume and constant surface area sets. This dual affiliation for spherical target was purposely constructed to link two different categories of plans. It is important to point out that the values presented in Fig 1 and Table 3 are to a great extent dependent on the underlying quality of plans. It took extra efforts to accomplish the stated treatment planning goals and to generate equivalent high-grade plans which provided meaningful data for comparisons. Throughout the planning process it became apparent that a suboptimal plan can produce larger irradiated brain volumes than presented in Fig 1 for plans of comparable quality.

The limitations of the study included selection bias inherent to retrospective approach, single-institution experience and lack of quantifiable toxicity data. The large majority of the selected cohort were low grade meningiomas. In general, the literature focusing on Gamma Knife meningioma radiosurgery are in accord that treatments are safe, effective and well tolerated[29]. Moreover, the symptomatic complications are subjective, difficult to scientifically quantify and typically transient. Although the overarching goal of the paper was to investigate surface area as an independent metric of normal tissue toxicity probability, due to the low incidence of radionecrosis in this cohort, no clinical predictions could be made based on surface area metrics alone. Furthermore, there are currently no prospectively validated metrics for dosimetric predictors of radionecrosis. The most commonly used and QUANTEC endorsed metric has been V_12Gy_, which was used in this study as a benchmark complication predictor[12, 21]. Ultimately, information gained in this study could be used to assess radionecrosis risk in other conditions in which the number of events is high enough for statistical association.

The phantom study and the linear regression analysis of clinical dataset offer several take home messages.

First, for the same target volumes, as the target surface area increases, the corresponding irradiated normal brain volumes V_12Gy_, V_14Gy_ and V_16Gy_ also increase. This is a direct consequence of the isoperimetric inequality which ascertains that a spherical target has the minimal surface area for a given volume.

Second, the constant target volume scenario provides a valuable insight why two patients with equal tumor volumes, and all other treatment parameters identical, can still exhibit different normal tissue complication probabilities. Diverse and complex patient specific biological circumstances present an attractive possible explanation. However, the simplest explanation for an adverse effect, or the lack of it, could be the variance in surface area of two targets.

Third, for the same target surface areas, a conventional wisdom is confirmed, as the target volume increases, the corresponding irradiated normal brain volumes V_12Gy_, V_14Gy_ and V_16Gy_ also increase.

Forth, the constant target surface area scenario offers remarkably different view on toxicity which would supplement current clinical knowledge and experience. In an imaginable novel approach, radiotherapy targets would be characterized and stratified by surface area. Since target surface area is independent of plan quality an a priori maximum treatment toxicity could be estimated purely based on surface area measurements, before an actual treatment plan is even made.

As a final remark, considerations of target surface area could introduce a new avenue for further development of treatment planning dose optimization algorithms.

## Supporting information

S1 File Plan Parameters. (PDF)
