# Supplementary material for: Considerations of target surface area and the risk of radiosurgical toxicity
Source: PLoS One. 2019 Oct 21;14(10):e0224047. doi: 10.1371/journal.pone.0224047 (PMC6802845; doi:10.1371/journal.pone.0224047)
Supplement: S1 File — (PDF) [file pone.0224047.s001.pdf]

Plan	Coverage/ Selectivity	Gradient Index	Number of shots	Tumor Largest Dimension [cm]	Tumor Volume V_{LGP} [cm^3]	Tumor Surface Area [cm^2]	V_{12Gy} [cm^3]	V_{14Gy} [cm^3]	V_{12Gy}-V_{LGP} [cm^3]	V_{14Gy}-V_{LGP} [cm^3]	Age [years]	Sex	Tumor
1	98/76	2.62	29	2.79	6.1	23.0	10.9	8.8	4.7	2.7	60	F	R Petroclival
2	97/88	2.48	21	2.17	3.5	13.3	5.3	4.3	1.8	0.8	75	F	L Petrous
3	95/86	2.69	13	1.70	1.7	7.5	2.6	2.1	1.0	0.4	93	F	L Clinoid
4	95/63	3.02	13	2.79	0.6	6.9	1.5	1.1	0.8	0.5	55	F	R Cerebellopontine Angle
5	95/79	2.84	22	3.65	5.1	22.6	9.0	6.9	4.0	1.9	58	F	L Petroclival
6	95/82	2.64	5	1.32	0.7	4.5	1.2	0.9	0.4	0.2	66	F	L Petroclival
7	96/82	2.95	27	2.43	4.1	14.9	7.2	5.5	3.1	1.4	65	F	R Cerebellopontine Angle
8	97/66	2.98	4	1.35	0.7	4.7	1.7	1.3	0.9	0.5	75	F	R Parfalcine
9	98/84	2.61	36	3.74	9.9	32.6	16.1	12.9	6.2	2.9	81	M	R Cavernous Sinus
10	95/79	2.69	24	3.29	4.1	17.0	7.0	5.6	2.9	1.5	56	F	L Cavernous Sinus
11	99/58	2.84	11	1.34	0.8	5.8	2.0	1.5	1.2	0.7	70	F	R Middle Cranial Fossa
12	98/70	2.72	18	2.46	2.2	12.6	4.3	3.4	2.1	1.3	21	M	L Tentorial Incisura
13	97/72	2.74	13	1.90	1.6	9.9	3.0	2.4	1.5	0.8	69	F	L Trigeminal
14	100/63	2.56	10	1.72	1.2	7.2	2.6	2.1	1.4	0.9	70	M	L Middle Cranial Fossa
15	95/90	2.96	26	2.38	4.4	14.0	6.7	5.2	2.4	0.9	71	F	R Clinoidal
16	98/56	2.73	15	4.24	3.6	15.3	8.8	7.1	5.2	3.5	50	F	Superior Sagittal Sinus
17	99/61	2.68	13	1.44	0.9	6.6	2.2	1.7	1.2	0.8	72	F	R Frontal Convexity
18	95/81	2.65	30	3.18	3.6	15.4	6.0	4.8	2.4	1.2	74	F	R Cavernous Sinus
19	95/71	2.65	17	2.40	2.0	12.1	3.8	3.1	1.8	1.0	33	F	L Petrous
20	99/48	2.95	18	2.62	2.9	22.6	9.1	7.0	6.2	4.1	78	F	L Frontal Convexity
21	100/64	2.52	24	3.40	7.7	28.7	16.6	13.4	8.9	5.7	65	F	L Tentorial Posterial Fossa
22	97/88	2.65	20	3.41	8.6	25.0	13.4	10.7	4.8	2.1	75	F	L Parafalcine
23	97/80	2.61	20	2.95	5.1	19.2	8.4	6.7	3.4	1.7	35	F	R Meckel's Cave
24	99/51	2.68	59	4.65	4.6	34.9	12.6	10.0	8.0	5.4	66	M	L Cavernous Sinus
25	98/38	3.04	34	4.30	4.5	33.1	18.2	13.7	13.8	9.2	54	F	L Paramedian Falx
26	99/64	3.11	12	2.15	1.1	8.1	2.6	2.0	1.5	0.9	64	F	L Frontal Convexity
27	93/66	2.95	7	2.08	1.9	11.2	4.4	3.4	2.5	1.6	45	F	R Cavernous Sinus
28	99/53	2.67	8	1.60	0.6	5.4	1.7	1.4	1.1	0.7	77	M	L Falcine
29	100/71	2.79	11	1.78	1.0	5.8	2.0	1.6	1.0	0.6	46	F	Planum Sphenoid
30	98/68	2.59	14	3.04	4.0	14.7	8.0	6.5	4.1	2.5	73	F	R Frontal Convexity
31	98/63	2.64	19	2.78	4.1	18.4	8.9	7.2	4.8	3.1	48	M	L Falcine
32	100/47	2.84	10	1.67	0.8	6.9	2.5	1.9	1.7	1.1	70	F	L Sphenoid
33	96/91	2.89	33	2.41	4.6	16.9	7.3	5.7	2.7	1.1	66	F	L Tentorial
34	98/85	2.49	15	2.48	4.4	14.3	6.9	5.7	2.5	1.2	79	F	L Petrous
35	99/77	2.53	12	2.42	3.4	12.7	6.2	5.0	2.7	1.5	81	M	L Temporal Convexity
36	99/61	2.78	9	2.82	2.2	27.5	5.2	4.1	3.0	1.9	80	M	L Middle Cranial Fossa
37	99/65	2.71	46	5.50	11.5	47.6	26.3	20.9	14.7	9.3	66	M	L Superior Sagittal Sinus
38	99/74	2.97	20	2.28	2.4	11.0	4.9	3.8	2.5	1.4	76	M	L Frontal Convexity
39	96/67	3.39	41	4.96	12.6	96.6	30.1	22.1	17.6	9.5	62	F	Superior Sagittal Sinus
40	99/48	2.75	12	1.66	0.7	6.4	1.9	1.5	1.3	0.9	52	F	L Petrous
41	100/58	2.78	33	5.68	6.9	52.1	17.1	13.4	10.2	6.5	42	F	Superior Sagittal Sinus
42	97/49	2.53	3	1.45	0.7	5.8	1.8	1.5	1.1	0.8	59	F	L Clinoidal
43	98/84	3.02	11	2.36	4.6	13.7	8.0	6.2	3.4	1.5	73	F	R Posterior Fossa
44	99/61	2.66	39	5.90	12.0	83.3	27.9	22.4	15.9	10.4	38	F	Superior Sagittal Sinus
45	99/70	2.88	9	1.80	1.2	6.6	2.6	2.0	1.4	0.8	44	F	R Orbital
46	98/73	2.88	15	2.42	3.8	15.8	7.5	5.8	3.7	2.0	61	F	R Middle Cranial Fossa
47	99/73	2.72	13	2.00	2.2	9.3	4.4	3.5	2.1	1.2	53	F	R Anterior Falcine
48	99/52	2.88	17	2.68	2.6	13.6	7.1	5.6	4.5	3.0	64	F	L Tentorial
49	96/83	2.67	9	2.00	2.1	8.4	3.6	2.9	1.5	0.7	61	F	R Superior Saggital Sinus
50	97/72	2.84	6	1.08	0.4	2.8	0.8	0.6	0.4	0.2	54	F	L Planum Sphenoid
51	99/63	2.72	24	5.43	9.1	46.7	20.4	16.3	11.4	7.2	72	M	Superior Sagittal Sinus

52	100/71	2.73	15	2.08	1.4	7.1	2.8	2.2	1.4	0.8	57	F	Frontal Parafalcine
53	96/65	3.22	76	9.02	12.1	57.1	29.5	22.3	17.3	10.1	62	M	Superior Sagittal Sinus
54	96/86	2.96	20	2.10	2.9	11.8	5.0	3.8	2.1	0.9	48	F	L Cerebellopontine Angle
55	98/81	2.86	36	2.35	2.7	12.5	4.7	3.7	2.0	1.0	53	F	R Cerebellopontine Angle
56	98/77	2.51	15	2.30	2.5	11.3	4.4	3.6	1.9	1.1	60	M	R Superior Sagittal Sinus
57	98/81	4.18	20	2.20	2.6	10.9	5.7	3.9	3.1	1.3	61	F	L Tentorial
58	99/75	2.98	24	2.22	1.4	9.0	2.8	2.1	1.3	0.7	47	F	L Falcine
59	99/73	2.61	11	1.96	2.2	9.2	4.1	3.3	1.9	1.1	48	F	R Torcular
60	99/69	2.64	15	2.66	2.8	14.4	5.7	4.5	2.9	1.7	41	F	L Frontal Parasagittal
61	97/78	2.72	10	2.44	3.0	13.2	5.5	4.4	2.5	1.3	65	M	L Occipital
62	98/62	2.8	16	2.08	1.4	16.0	3.3	2.6	1.9	1.2	57	F	R Tentorial
63	97/87	2.9	13	2.62	4.6	15.2	7.3	5.8	2.8	1.2	69	M	L Tentorial
64	98/66	2.85	20	3.34	2.4	31.4	5.1	4.1	2.7	1.6	59	F	L Superior Sagittal Sinus
65	97/84	2.64	14	3.84	14.4	43.2	23.4	18.9	9.1	4.5	70	M	R Frontal Convexity
66	99/73	2.92	9	1.96	1.1	6.6	2.3	1.8	1.1	0.7	54	F	R Tentorial Incisura
67	99/76	2.82	11	1.60	0.8	4.9	1.5	1.2	0.7	0.4	55	F	L Cavernous Sinus
68	99/68	3.21	13	2.15	2.5	12.2	6.3	4.5	3.8	2.0	44	F	R Temporal Convexity
69	99/74	2.85	5	1.26	0.6	3.7	1.2	0.9	0.6	0.3	62	F	R Falcine
70	97/88	2.64	13	1.60	1.6	6.9	2.6	2.0	0.9	0.4	71	F	R Posterior Falcine
71	98/71	2.5	9	2.14	1.1	5.8	2.1	1.7	1.0	0.6	54	F	Superior Sagittal Sinus
72	96/91	2.65	27	2.68	2.9	12.2	4.7	3.8	1.8	0.9	65	F	L Petrous
73	98/75	2.78	24	2.33	2.7	13.6	5.2	4.1	2.5	1.4	37	F	L Falcine
74	98/84	2.83	18	1.72	1.2	14.3	2.1	1.7	0.9	0.4	50	F	R Falcine
75	96/95	2.41	24	3.26	12.7	30.7	17.6	14.6	4.9	1.8	78	F	L Frontal Parafalcine Convexity
76	98/72	2.75	24	3.38	5.1	26.1	9.9	7.8	4.8	2.7	47	M	R Meckel's Cave
77	97/84	2.5	26	3.14	9.9	29.0	15.2	12.5	5.4	2.7	84	F	R Sigmoid Sinus
78	99/84	2.59	21	2.32	3.6	12.6	5.9	4.7	2.4	1.2	71	F	R Petrous
79	98/81	2.64	16	1.69	1.7	8.0	2.9	2.3	1.2	0.6	81	F	R Cerebellopontine Angle
80	98/60	2.95	8	2.65	1.9	8.5	4.6	3.6	2.8	1.7	31	F	R Superior Sagittal Sinus
81	97/62	2.89	19	3.45	3.7	52.9	8.5	6.6	4.8	2.9	62	F	R Tentorial
82	96/88	2.57	15	2.40	3.4	12.9	5.2	4.2	1.8	0.9	69	M	R Tentorial
83	98/81	3.3	15	2.57	4.3	13.9	8.2	6.1	3.9	1.9	52	M	L Tentorial
84	98/80	2.89	11	1.92	1.9	8.3	3.6	2.8	1.6	0.8	53	F	R Superior Sagittal Sinus
85	93/91	2.66	23	2.48	5.6	17.6	8.3	6.6	2.8	1.0	66	F	R Frontal Convexity
86	99/87	2.59	14	1.95	2.4	9.3	3.7	3.0	1.4	0.7	58	M	Posterior Falcine
87	98/75	2.81	21	5.00	20.3	58.8	39.1	30.9	18.9	10.6	67	F	R Cavernous Sinus
88	97/75	2.51	20	2.70	6.5	19.5	10.2	8.3	3.7	1.8	54	F	R Frontal Parafalcine
89	94/82	2.87	25	4.58	14.7	53.3	25.0	19.5	10.3	4.9	71	M	R Cavernous Sinus
90	96/84	2.89	28	2.04	1.9	9.8	3.2	2.5	1.3	0.6	59	F	L Cerebellopontine Angle
91	96/82	2.65	19	2.38	3.8	14.2	6.2	5.0	2.4	1.2	64	F	R Cavernous Sinus
92	96/71	2.88	22	5.66	5.8	34.7	11.6	9.2	5.8	3.4	44	M	R Superior Sagittal Sinus
93	97/90	2.58	19	2.92	8.1	22.0	12.0	9.7	3.9	1.7	54	F	R Transverse Sinus
94	98/69	2.59	10	2.11	2.5	10.0	5.1	4.1	2.6	1.6	56	F	L Transverse Sinus
95	96/69	3.1	45	4.24	6.7	41.1	14.5	11.0	7.8	4.4	31	F	L Superior Sagittal Sinus
96	99/69	2.83	13	2.61	1.7	17.1	3.7	2.9	1.9	1.1	48	F	R Superior Sagittal Sinus
97	96/65	3.14	26	4.04	3.7	29.1	8.4	6.3	4.8	2.7	69	F	R Cavernous Sinus
98	97/72	2.62	20	2.36	4.3	17.0	8.1	6.5	3.9	2.3	46	M	R Clinoid
99	92/55	3.27	51	4.95	3.0	24.8	8.5	6.4	5.6	3.4	53	F	L Cavernous Sinus
100	97/85	2.65	19	1.88	2.2	8.9	3.7	2.9	1.4	0.7	59	F	R Transverse Sinus
